# Anisotropic magnetic property, magnetostriction, and giant magnetocaloric effect with plateau behavior in TbMn_2_Ge_2_ single crystal

**DOI:** 10.1038/s41598-022-23661-4

**Published:** 2022-11-04

**Authors:** Shuai Huang, Yuming Bai, Kaiqi Wan, Changming Zhu, Dexuan Huo, Zhaoming Tian

**Affiliations:** 1 Key Laboratory of Novel Materials for Sensor of Zhejiang Province, Institute of Material Physics, Hangzhou Dianzi University, Hangzhou, 310018 People’s Republic of China; 2 School of Physics and Wuhan National High Magnetic Field Cent, Huazhong University of Science and Technology, Wuhan, 430074 People’s Republic of China; 3 Guangxi Key Laboratory of Nuclear Physics and Nuclear Technology, Guangxi Normal University, Guilin, 541004 People’s Republic of China

**Keywords:** Magnetic properties and materials, Ferromagnetism

## Abstract

The ternary RMn_2_Ge_2_ (R = rare earth) intermetallic compounds have attracted great attention due to their interesting magnetic behaviors and magnetotransport responses. Here, we reported our observation of anisotropic magnetic property, magnetostriction, and magnetocaloric effect (MCE) in TbMn_2_Ge_2_ single crystal. Below the transition temperature of Tb magnetic sublattices ($T_{{\text{C}}}^{{{\text{Tb}}}}$ ~ 95 K), strong Ising-like magnetocrystalline anisotropy is observed with an out-of-plane ferromagnetic moments 5.98 *μ*_B_/f.u. along the easy *c* axis, which is two orders of magnitude larger than that of field along *a* axis. Above $T_{{\text{C}}}^{{{\text{Tb}}}}$, a field-induced metamagnetic transition is observed from the spin-flip of Mn sublattices. During this transition, remarkable magnetostriction effect is observed, indicating of strong spin–lattice coupling. The responses of Tb and Mn sublattices to the magnetic field generate a giant magnetic entropy change ($- \Delta S_{M}$) and large values of relative cooling power (RCP) and temperature-averaged entropy change (TEC). The calculated maximum magnetic entropy change ($- \Delta S_{M}^{\max }$), RCP, and TEC(10) with magnetic field change of 7 T along *c* axis reach 24.02 J kg^−1^ K^−1^, 378.4 J kg^−1^, and 21.39 J kg^−1^ K^−1^ near $T_{{\text{C}}}^{{{\text{Tb}}}}$, which is the largest among RMn_2_Ge_2_ families. More importantly, this giant MCE shows plateau behavior with wide window temperatures from 93 to 108 K, making it be an attractive candidate for magnetic refrigeration applications.

## Introduction

Magnetocaloric effect (MCE) is the phenomenon which converts magnetic energy into thermal energy by changing the applied magnetic field^1–6^. Based on the MCE, magnetic refrigeration technology is proposed, and it has attracted great attention due to the high energy efficiency and environmental friendliness. The MCE can be quantified by adiabatic temperature change or isothermal magnetic entropy change ($- \Delta S_{M}$) for a certain magnetic field variation^1–6^. Up to date, numerous magnetic refrigeration materials with giant $- \Delta S_{M}$ accompanied by first order phase transition have been reported^5,6^. Although the MCE is intrinsic for all magnetic materials, only some of them which have strong MCE and small hysteresis loss are desirable for practical application^1^. In addition, giant reversible MCE only appears in a narrow temperature range for most of the existing magnetic refrigeration materials. Therefore, it is important to search for materials that exhibit giant MCE not only with small hysteresis loss but also with a wide temperature range.

**Figure 1:**
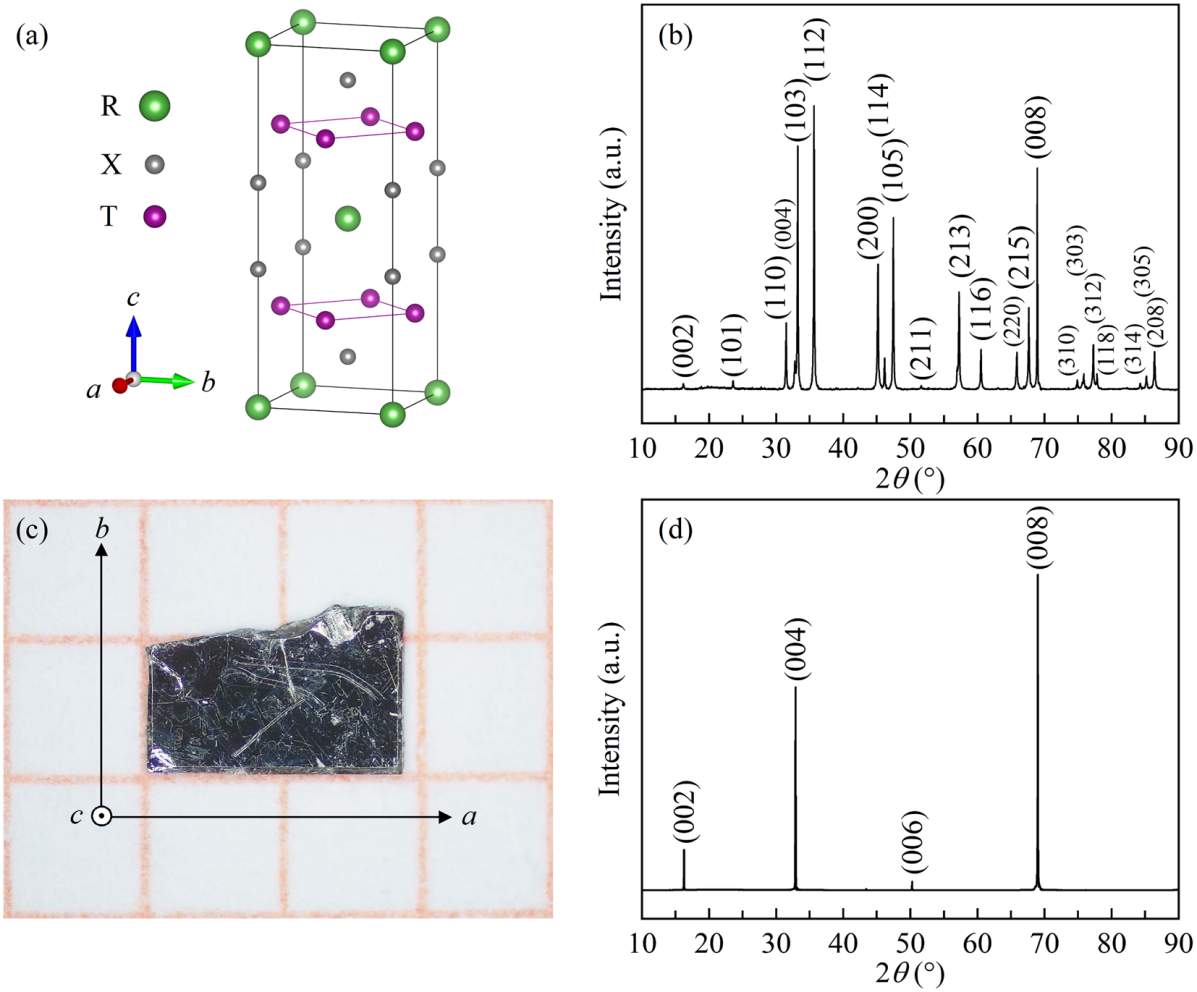
(**a**) The crystal structure of RT_2_X_2_ compounds. The green, grey, and purple balls represent the R, X, and T atoms, respectively. (**b**) Room-temperature powder XRD patterns of TbMn_2_Ge_2_. (**c**) The representative photograph of TbMn_2_Ge_2_ single crystal. (**d**) Room-temperature XRD patterns for TbMn_2_Ge_2_ single crystal recorded on (00*l*) plane.

The ternary intermetallic compounds with the formula of RT_2_X_2_ (R = rare earth, T = transition metal, and X = Si, Ge) have been extensively studied due to superconductivity, magnetic ordering, and heavy-fermion properties^7^. During the last few years, the compounds have also been found to possess giant MCE with small hysteresis loos near the magnetic ordering temperature^8–12^. They crystalize in ThCr_2_Si_2_-type tetragonal structure with space group *I*4/*mmm*^7^. As shown in Fig. 1a, the structure consists of R, X, and T layers which alternately stack along *c* axis, and the R, T, and X atoms occupy 2*a*, 4*d*, and 4*e* positions, respectively^7^. In this series, special attention has been devoted to RMn_2_X_2_ compounds because of the magnetic Mn sublattices^11–38^. They present a vast variety of magnetic structures and magnetic phase transitions, and the magnetic state can be selected by controlling the interlay and intralayer distance between the magnetic atoms^21–23^. Very recently, the RMn_2_Ge_2_ compounds have triggered renewed interests due to the nontrivial transport behaviors, such as the spontaneous skyrmions and the giant topological Hall effect^39–45^. From the results of magnetization measurement in single crystal samples, significant change in magnetization is obtained during the magnetic transition. For example, a spin reorientation of Mn moments is observed at 215 K for NdMn_2_Ge_2_^40^, and a rapid upturn of magnetic susceptibility from paramagnetic (PM) to ferromagnetic (FM) transition along *c* axis is obtained at 325 K^39^. These features suggest that the giant MCE may be available near the magnetic transition region. Additionally, a magnetic field-induced metamagnetic transition at certain temperature has been detected in Tb_1-*x*_Y_*x*_Mn_2_Ge_2_^33^. As the direction of the magnetic moment and the symmetry of the spin texture in metamagnetic transition can be easily changed by external magnetic field, sufficient low field MCE can be achieved^12^. To clarify the intrinsic magnetic behavior and the possible application in magnetic refrigeration, it is important to investigate the magnetic properties on single crystal samples. In this work, we present a systematic study on the anisotropy magnetic property, magnetostriction, and the MCE in TbMn_2_Ge_2_ single crystal. Large magnetocrystalline anisotropy below the transition temperature of Tb magnetic sublattices ($T_{{\text{C}}}^{{{\text{Tb}}}}$ ~ 95 K) and a field-induced metamagnetic transition above $T_{{\text{C}}}^{{{\text{Tb}}}}$ are observed, and remarkable magnetostriction is accompanied by these magnetic transitions. Strikingly, giant MCE with plateau behavior in a wide window temperature near $T_{{\text{C}}}^{{{\text{Tb}}}}$ is obtained for magnetic field along *c* axis. Moreover, a magnetic phase diagram is constructed based on the magnetic results.

## Experimental details

The TbMn_2_Ge_2_ single crystal was grown using an Indium flux method^39,42^. The starting materials with a molar ratio of Tb:Mn:Ge:In = 1:2:2:20 were put into an alumina crucible and sealed in a quartz tube under high vacuum. During the growth process, the tube was firstly heated up to 1373 K and kept at this temperature for 12 h. Then, it was slowly cooled to 973 K with a rate of 2 K/h. Finally, to separate the single crystal from the Indium flux, the tube was taken out from the furnace at 973 K, and the Indium flux was decanted by centrifugation.

The crystal structure was checked using an x-ray diffractometer (XRD, SmartLab, Rigaku) with Cu-*K*_*α*_ radiation. The crystal orientation was checked using a Rigaku XtaLab-mini-II diffractometer with Mo-*K*_*α*_ radiation. The elemental analysis was performed on a scanning electron microscope (SEM, JSM-IT500HR, JEOL) with an energy dispersive spectrometer (EDS). The magnetic measurements were carried out using a vibrating sample magnetometer (VSM) on a physical property measurement system (PPMS, Quantum Design). The isothermal magnetization curves for calculating the $- \Delta S_{M}$ were measured in discrete steps from 90 to 115 K with temperature interval of 0.5 K, and the sample was heated to the PM temperature region and demagnetized in oscillatory mode before each measurement^46^. To eliminate the influence of demagnetizing field, the demagnetization correction was performed, and the effective field was estimated by subtracting the demagnetization field from the external field (*μ*_0_*H*_ext_) using the equation $\mu_{0} H = \mu_{0} (H_{{{\text{ext}}}} - N_{{\text{d}}} M)$, where *N*_d_ is the demagnetization factor^47^. The *N*_d_ is calculated to be 0.1085 and 0.6629 along *a* and *c* axes, respectively. All the results were discussed based on the data after the demagnetization correction. The magnetostriction was measured using a strain gauge (KFLB, Kyowa) based on Wheatstone bridge principle, and the imbalance of the bridge was measured using a lock-in amplifier (SR830, Stanford Research Systems).

## Results and discussion

The room-temperature powder XRD pattern of TbMn_2_Ge_2_ is shown in Fig. 1b. The diffraction peaks can be well fitted with the tetragonal structure^7^, which confirms high purity of the prepared sample. The stoichiometry ratio of Tb, Mn, and Ge is estimated to be 20.82:39.47:39.71 from the EDS results, indicating the spatially uniform stoichiometry of TbMn_2_Ge_2_ single crystal (see Fig. S1 in supplementary material for details). Figure 1c shows the representative photograph of TbMn_2_Ge_2_ single crystal on the grid paper. The crystal has a rectangular shape in a dimension of 1.88 × 0.92 × 0.29 mm^3^. Room-temperature XRD experiment was performed on the plate-like single sample. As shown in Fig. 1d, the XRD patterns for the front of the plate can be indexed by the indices of (00* l*) (*l* = 2, 4, 6, 8) plane, and the patterns for the side are indexed by the indices of (*h*00) (*h* = 2) plane (see Fig. S2 in supplementary material for details). The results indicate that the in-plane and out-of-plane directions are parallel and perpendicular to the *ab* plane. The directions of *a* and *b* axes are indicated by the arrows in Fig. 1c, and the normal direction of the plate is parallel to *c* axis.

**Figure 2:**
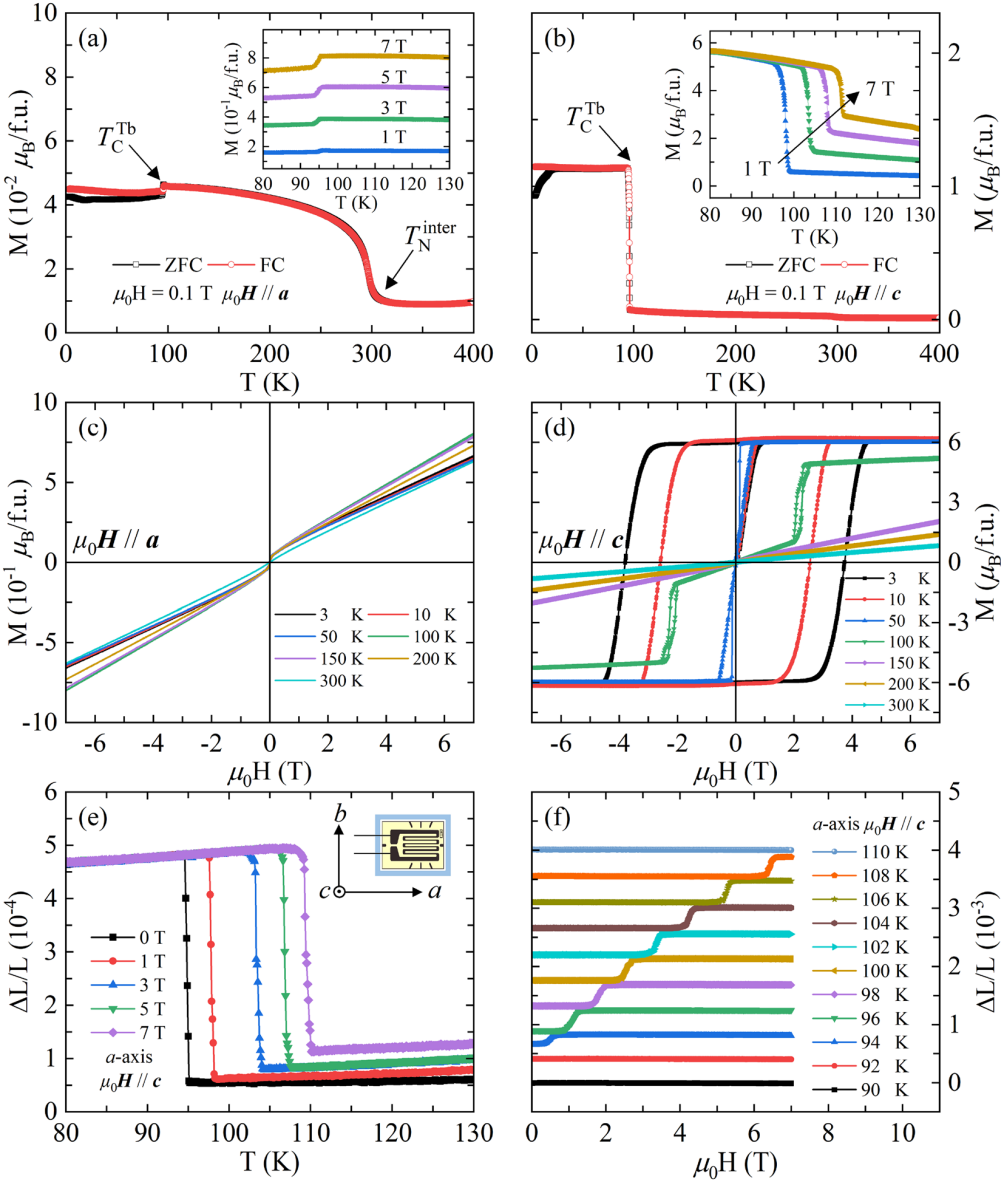
Temperature dependence of ZFC and FC magnetization measured with *μ*_0_H = 0.1 T along (**a**) *a* and (**b**) *c* axes. The insets of (**a**) and (**b**) show the FC curves with various magnetic fields near $T_{{\text{C}}}^{{{\text{Tb}}}}$. (**c**) and (**d**) show the isothermal magnetic hysteresis loops from 3 to 300 K with *μ*_0_*H*//*a* and *μ*_0_*H*//*c*, respectively. (**e**) Temperature dependence of the strain change along *a* axis with *μ*_0_*H*//*c*. The inset shows the schematic of the strain gauge and the sample. (**f**) Magnetic field dependence of magnetostriction [ΔL/L = ΔL/L(H) − ΔL/L(0)] along *a* axis with *μ*_0_*H*//*c* at different temperatures. For clarity, the curves are shifted upward except for the result with T = 90 K.

To explore the magnetic properties, temperature dependence of zero-field-cooled (ZFC) and field-cooled (FC) magnetization with *μ*_0_H = 0.1 T along *a* and *c* axes is measured in the temperature range from 3 to 400 K, and the results are shown in Fig. 2a,b, respectively. With decreasing temperature, the magnetic behavior is quite different for magnetic field along *a* and *c* axes, exhibiting large magnetocrystalline anisotropy. For *μ*_0_*H*//*a* in Fig. 2a, there are two remarkable transitions, which include a gradual increase in magnetization at 296 K and a sudden decrease at 95 K. While for *μ*_0_*H*//*c* in Fig. 2b, an abrupt increase in magnetism at 95 K is observed. Based on the neutron diffraction experiment, L. Morellon, *et*. *al*. have demonstrated that the moments of Mn atoms are FM ordering along *c* axis and the Tb moments are also ordered ferromagnetically in the same direction but antiferromagnetically to the Mn sublattices, yielding a collinear ferrimagnetic (Ferri) spin structure along *c* axis below $T_{{\text{C}}}^{{{\text{Tb}}}}$^19–21^. Our results agree well with the neutron diffraction data, but a slightly lower $T_{{\text{C}}}^{{{\text{Tb}}}}$ ~ 95 K is found. The insets of Fig. 2a,b show the FC curves measured with various magnetic fields near $T_{{\text{C}}}^{{{\text{Tb}}}}$. With the increase of magnetic field, the transition temperature is driven to higher temperature for *μ*_0_*H*//*c*, while it remains unchanged for *μ*_0_*H*//*a*. For the transition at 296 K along *a* axis, although there is no change in the spin configuration between 200 and 400 K according to the neutron diffraction experiments, similar behavior is observed in Pr_1-*x*_Tb_*x*_Mn_2_Ge_2_^19^. It is reported that an AFil-type spin structure is formed below Néel temperature ($T_{{\text{N}}}^{{{\text{inter}}}}$), and the moments of Mn are FM ordering within the *ab* layers but antiferromagnetic (AFM) coupling along *c* axis at $T_{{\text{C}}}^{{{\text{Tb}}}}$ < T < $T_{{\text{N}}}^{{{\text{inter}}}}$^21^. The FM component of Mn moments in the *ab* layers may be response for the FM-like transition at 296 K^48^.

Figure 2c,d show the magnetic field dependence of magnetization in TbMn_2_Ge_2_ single crystal with *μ*_0_*H*//*a* and *μ*_0_*H*//*c*, respectively. The magnetization increases almost linearly with increasing the magnetic field along *a* axis, and the isothermal magnetization curves show negligible hysteresis. The hysteresis loops along *c* axis exhibit FM nature for T < 100 K, and typical hysteresis is observed at 3 and 10 K. It is reported that the magnetic phase is not changed below ^19,21^. However, we note that the ZFC and FC curves deviate from each other below ~ 20 K (Fig. 2b), and similar phenomenon is observed in previous works^19,30,36^, suggesting the competition between different magnetic properties. J. Leciejewicz et al. have found that the Tb magnetic sublattice transforms into an AFM one below 26 K^32^. Additionally, it is worth noting that the similar system TbMn_2_Si_2_ exhibits a canted Ferri structure in which the Tb moments lie along the *c* axis, and the Mn moments are parallel with each other in their own layer and are canted between the Mn sublayers^11^. The possible changes in magnetic structure may be response for the large coercive field for the data measured at 3 and 10 K in Fig. 2d. The saturation magnetization reaches 5.98 *μ*_B_/f.u., which is slightly larger than the previous reported value (5.53 *μ*_B_/f.u.)^20,21^. At 100 K, the hysteresis loop is linear with the increase of magnetic field at first, and then the magnetic moment is fully polarized at a critical field (*μ*_0_*H*_C_) of 2.4 T, indicating field-induced metamagnetic transition. Due to that the spin of Tb atoms is disordered above $T_{{\text{C}}}^{{{\text{Tb}}}}$, the metamagnetic transition should result from the spin-flip of Mn. Before the metamagnetic transition, Mn magnetic moments are AFM ordering along *c* axis^19,21^. When the magnetic field is larger than the *μ*_0_*H*_C_, the FM ordering of Mn moments is formed. At T ≥ 150 K, linear hysteresis loops are observed due to the AFM interaction of Mn^20^.

To investigate the possible coupling between the magnetic ordering and the structural parameter, the strain change is measured around the magnetic transition region. As shown in Fig. 2e, a sharp step in the relative length change (ΔL/L) is observed along *a* axis at $T_{{\text{C}}}^{{{\text{Tb}}}}$. When the field is applied along the easy magnetization *c* axis, the step moves to higher temperature, and it coincides with the magnetic transition in the inset of Fig. 2b. Based on the Rietveld analysis of neutron diffraction spectra^21^, it is found that a variation of the Mn magnetic moment, Δ*μ*_Mn_/*μ*_Mn_ ≈16%, takes place at $T_{{\text{C}}}^{{{\text{Tb}}}}$. This transition causes a dramatic jump in *a* cell parameter, Δ*a*/*a* ≈ 0.15%, and no anomaly is found in *c* cell parameter. As we know, the spin alignment of adjacent Mn layers is antiparallel along *c* axis when $T_{{\text{C}}}^{{{\text{Tb}}}}$ < T < $T_{{\text{N}}}^{{{\text{inter}}}}$, and it becomes parallel along *c* axis below $T_{{\text{C}}}^{{{\text{Tb}}}}$. It means that the value of *a* cell parameter is correlated with the spin configuration of Mn. When the magnetic field is applied along *c* axis, the metamagnetic transition results in the change of Mn spin configuration. This process would induce the anomaly in *a* cell parameter. Our results agree well with the data in polycrystalline TbMn_2_Ge_2_^21,23^. The field dependence of ΔL/L along *a* axis with *μ*_0_*H*//*c* is shown in Fig. 2f. It is obvious that a visible change of ΔL/L is observed at a critical field ($\mu_{0} H^{\prime}_{{\text{C}}}$), and the $\mu_{0} H^{\prime}_{{\text{C}}}$ increases with increasing temperature, suggesting remarkable magnetostriction effect. When the magnetic field is applied along *a* axis, the field-induced anomalies are unavailable (see Fig. S3 in supplementary material for details). The strong response of the lattice to the external magnetic field indicates an unusually large coupling of the Mn magnetic moments to the lattice.

**Figure 3:**
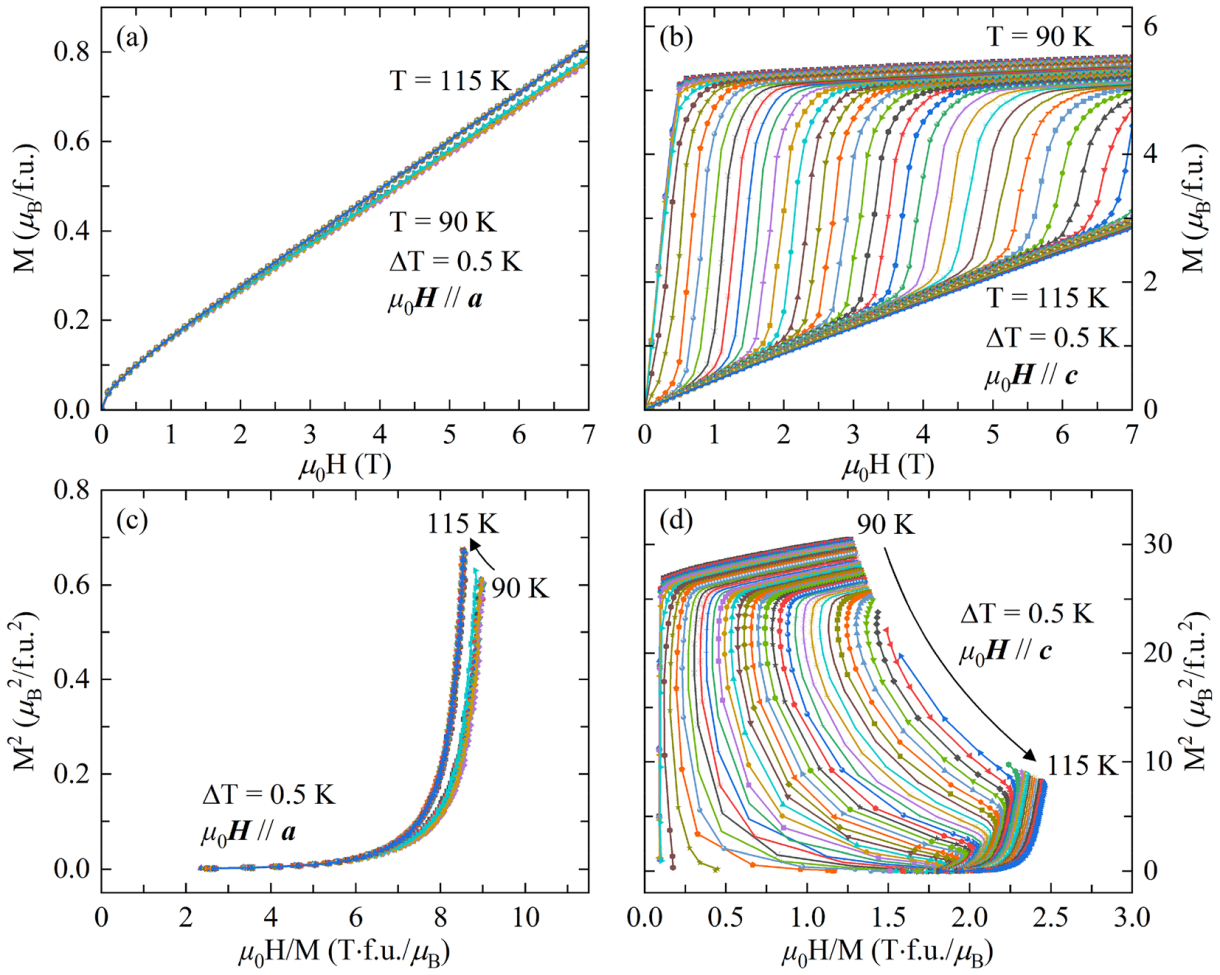
Isothermal initial magnetization curves in the temperature range from 90 to 115 K with an interval temperature of 0.5 K along (**a**) *a* and (**b**) *c* axes. (**c**) and (**d**) show the Arrott plot for magnetic field along *a* and *c* axes, respectively.

As significant changes in magnetization are obtained near the magnetic transition region in TbMn_2_Ge_2_ single crystal, giant MCE is expected, especially for *μ*_0_*H*//*c* axis. It is well known that giant MCE have a strong correlation with the first order magnetic phase transition^5^, so it is necessary to understand the nature of the magnetic transition in TbMn_2_Ge_2_ single crystal. Figure 3a,b show the isothermal initial magnetization curves with the magnetic field along *a* and *c* axes from 90 to 115 K with an interval temperature of 0.5 K. For *μ*_0_*H*//*a*, a continuous increase in the magnetization is observed at each temperature, and the magnetization increases slightly with the increase of temperature (Fig. 3a). While for *μ*_0_*H*//*c*, the magnetization gets saturated below 0.5 T magnetic field at 90 K (Fig. 3b). When the temperature reaches 95 K, the metamagnetic transition emerges. At lower temperature, the AFM anisotropy is weak and only a small magnetic field is required to fully polarize the spins along *c* axis. The *μ*_0_*H*_C_ of the metamagnetic transition gradually increases with the increase of temperature. According to Banerjee criterion, the magnetic transition belongs to second order if all the *μ*_0_H/M *vs* M^2^ curves (also named as Arrott plot) have positive slope. On the other hand, if some of the *μ*_0_H/M *vs* M^2^ curves show negative slope at some points, the magnetic transition is of the first order^49^. The Arrott plot for *μ*_0_*H*//*a* and *μ*_0_*H*//*c* are shown in Fig. 3c,d, respectively. Clearly, negative slope can be observed in the Arrott plot in Fig. 3d, indicating the occurrence of first order magnetic transition.

Based on the classical thermodynamical and the Maxwell thermodynamic relations, the $- \Delta S_{M}$ is given by^4^:

1 $$\Delta S_{M} = \int_{0}^{H} {\left[ {\partial S(T,H)/\partial H} \right]}_{T} dH = \int_{0}^{H} {\left[ {\partial M(T,H)/\partial T} \right]}_{H} dH,$$

where $[\partial S(T,H)/\partial H]_{T} = [\partial M(T,H)/\partial T]_{H}$ is based on the Maxwell relation. For magnetization measured at small temperature and field intervals, Eq. (1) can be rewritten as:

2 $$\Delta S_{M} = \frac{{\int_{0}^{H} {M(T_{i + 1} ,H)dH - \int_{0}^{H} {M(T_{i} ,H)dH} } }}{{T_{i + 1} - T_{i} }}.$$

**Figure 4:**
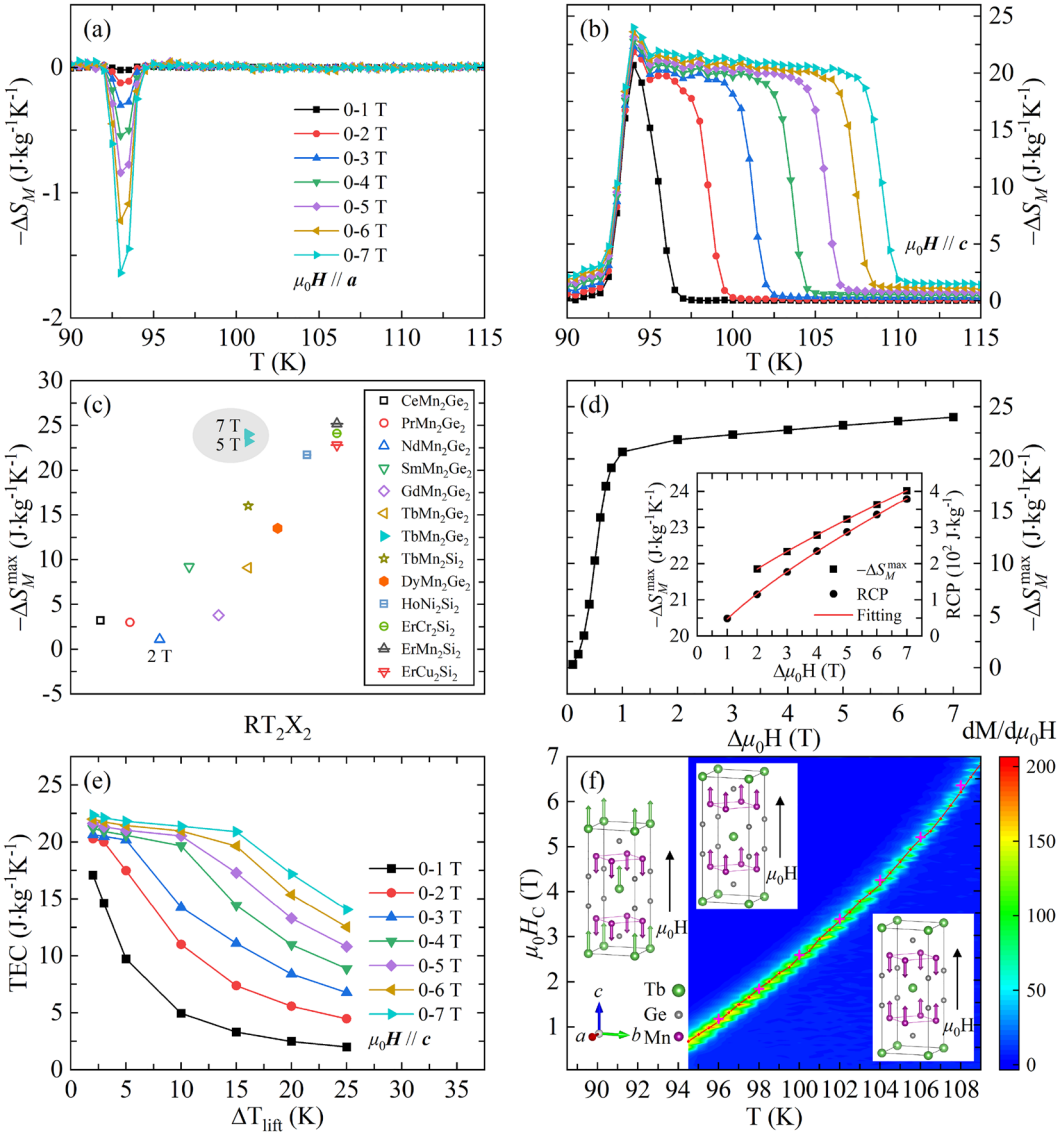
Temperature dependence of $- \Delta S_{M}$ for TbMn_2_Ge_2_ single crystal calculated from the isothermal initial magnetization along (**a**) *a* and (**b**) *c* axes. (**c**) Bubble chart of $- \Delta S_{M}^{\max }$ for the selected RT_2_X_2_ compounds. The solid and open symbols represent single crystal and polycrystal samples, respectively. The samples are arranged from left to right according to the atomic number of the rare earth elements in ascending order. The data in the dash area indicate our results. The Δ*μ*_0_H is 5 T if not specifically indicated. (**d**) The Δ*μ*_0_H dependence of $- \Delta S_{M}^{\max }$ for *μ*_0_*H*//*c*. The inset shows the field-dependent parameters of $- \Delta S_{M}^{\max }$ and RCP for *μ*_0_*H*//*c* with larger magnetic field, and the red curves show the nonlinear fitting based on a power law. (**e**) The TEC as a function of ΔT_lift_ for the field along *c* axis. (**f**) Magnetic phase diagram of TbMn_2_Ge_2_ single crystal for *μ*_0_*H*//*c*. The red balls denote the *μ*_0_*H*_C_ obtained from the isothermal curves transition, and the purple crosses indicate the ones from the magnetostriction results. The insets show the moments of Mn and Tb sublattices at specific magnetic field and temperature regions.

The temperature dependence of the calculated $- \Delta S_{M}$ using Eq. (2) with various magnetic field changes (Δ*μ*_0_H) along *a* and *c* axes are shown in Fig. 4a,b, respectively. For *μ*_0_*H*//*a*, an inverse MCE with maximum magnetic entropy change ($- \Delta S_{M}^{\max }$) of − 1.64 J kg^−1^ K^−1^ is obtained near $T_{{\text{C}}}^{{{\text{Tb}}}}$, and the inverse MCE could be attributed to the magnetic transition of Tb sublattices from disorder to order along *c* axis, which induces a slight increase of magnetization along *a* axis. In contrast, a normal MCE is obtained for the magnetic field along *c* axis, and the $- \Delta S_{M}^{\max }$ is evaluated to be 24.02 J kg^−1^ K^−1^ with Δ*μ*_0_H = 7 T at $T_{{\text{C}}}^{{{\text{Tb}}}}$, exhibiting giant MCE. The other significant feature in Fig. 4b is that a plateau behavior for the giant MCE is obtained with larger Δ*μ*_0_H, resulting in a wide temperature range with large $- \Delta S_{M}$. The peak width determined from the difference between the extreme values in the ${\text{d}}\Delta S_{M} {\text{/dT}}$ *vs* T curves is proportional to Δ*μ*_0_H, and it reaches up to 16 K with Δ*μ*_0_H = 7 T. For Δ*μ*_0_H = 5 T, the $- \Delta S_{M}^{\max }$ is evaluated to be 23.23 J kg^−1^ K^−1^. As shown in Fig. 4c, this value is much larger than that in TbMn_2_Ge_2_ polycrystal sample^33^ and other RMn_2_Ge_2_ compounds^14–18^. Meanwhile, it is comparable with the series of RT_2_X_2_ compounds with giant MCE^8–12^. Moreover, the temperature of $- \Delta S_{M}^{\max }$ appears at 95 K for present sample, which is much higher than the values in above-mentioned compounds.

Figure 4d shows the Δ*μ*_0_H dependence of $- \Delta S_{M}^{\max }$ for *μ*_0_*H*//*c*. The value of $- \Delta S_{M}^{\max }$ increases rapidly with increasing Δ*μ*_0_H from 0 to 1 T, and then it gets saturate gradually. For the relatively small value of Δ*μ*_0_H, the $- \Delta S_{M}^{\max }$ reaches up to 20.69 J kg^−1^ K^−1^ (Δ*μ*_0_H = 1 T), suggesting giant low field MCE. Except for the $- \Delta S_{M}$ parameter, the relative cooling power (RCP) is also usually used to characterize the potential MCE of materials, which indicates the amount of heat transfer between the cold and hot reservoirs in an ideal refrigeration cycle^6^. It is given by:

3 $${\text{RCP}} = - \Delta S_{M}^{\max } \delta T_{{{\text{FWHM}}}} ,$$

where FWHM means the full width at half maximum in $- \Delta S_{M}$ curve. The variation of RCP is consistent with that of $- \Delta S_{M}^{\max }$. As shown in the inset of Fig. 4d, for Δ*μ*_0_H larger than 1 T, both of $- \Delta S_{M}^{\max }$ and RCP parameters can be well fitted using the power law relations: $- \Delta S_{M}^{\max } = a + b(\mu_{0} H)^{m}$ and ${\text{RCP}} = c + d(\mu_{0} H)^{n}$, and the fitting yields the values of *a*, *b*, *m*, *c*, *d*, and *n* to be 20.52, 0.132, 0.770, − 61.14, 20.564, and 0.722. For TbMn_2_Ge_2_ single crystal, the RCP reaches a maximum value of 378.4 J kg^−1^ with Δ*μ*_0_H = 7 T along *c* axis, which is of the same order of magnitude as other high-performing magnetic refrigeration materials^11,12^. Considering that the RCP tends to overestimate the merit of materials, temperature-averaged entropy change (TEC) has recently been proposed as a more predictive figure of merit to identify the performance of the MCE materials, and it can be determined using the equation^50^:

4 $${\text{TEC}}(\Delta {\text{T}}_{{{\text{lift}}}} ) = \frac{1}{{\Delta {\text{T}}_{{{\text{lift}}}} }}\max \left\{ {\int\limits_{{{\text{T}}_{{{\text{mid}}}} - \frac{{\Delta {\text{T}}_{{{\text{lift}}}} }}{2}}}^{{{\text{T}}_{{{\text{mid}}}} + \frac{{\Delta {\text{T}}_{{{\text{lift}}}} }}{2}}} {\Delta S({\text{T}})_{{\Delta \mu_{0} {\text{H,T}}}} d{\text{T}}} } \right\},$$

where ΔT_lift_ is the desired temperature span of the device, and T_mid_ is the temperature at the center of the average that maximizes TEC(ΔT_lift_) for the given ΔT_lift_. Figure 4e shows the variation of TEC with ΔT_lift_ for the magnetic field along *c* axis. It is seen that the TEC decreases with increasing ΔT_lift_. The value of TEC(ΔT_lift_ = 10 K) increases continuously and reaches 21.39 J kg^−1^ K^−1^ with Δ*μ*_0_H = 7 T. The values of TEC(ΔT_lift_ = 3 and 10 K) are also comparable to the previous works, such as La(Fe_0.88_Si_0.12_)_13_^50^ and HoNiGe_3_^51^.

Based on above results, a phase diagram represented by the contour plot of dM/d*μ*_0_H *vs μ*_0_H curves is constructed for TbMn_2_Ge_2_ single crystal, and the data are shown in Fig. 4f. When $T_{{\text{C}}}^{{{\text{Tb}}}}$ < T < $T_{{\text{N}}}^{{{\text{inter}}}}$, the AFil-type magnetic structure is formed, and the spin alignment of adjacent Mn layers (interlayer) is antiparallel along *c* axis (right inset of Fig. 4f)^19–21^. Below $T_{{\text{C}}}^{{{\text{Tb}}}}$, the Tb atoms order ferromagnetically along *c* axis and antiferromagnetically to the Mn sublattices (left inset of Fig. 4f). The magnetic ordering of Tb atoms accompanied by the magnetostriction effect is the mainly origination of the giant MCE. Additionally, the fully polarized FM state of Mn sublattices is established above the *μ*_0_*H*_C_ when T > $T_{{\text{C}}}^{{{\text{Tb}}}}$ (the middle inset of Fig. 4f). The coincide of *μ*_0_*H*_C_ and $\mu_{0} H^{\prime}_{{\text{C}}}$ curves implies that the remarkable magnetostriction effect is mainly due to the field-induced metamagnetic transition. The magnetic transition from AFil to FM state of Mn sublattices leads to the plateau behavior of the $- \Delta S_{M}$ curves, and the giant MCE still retains without the ordering of Tb atoms. Further investigation of the magnetic structure evolution based on high magnetic field and neutron scattering measurement in TbMn_2_Ge_2_ single crystal is required to refine the phase diagram.

## Conclusions

IN summary, we have studied the anisotropic magnetic property, magnetostriction, and the MCE in TbMn_2_Ge_2_ single crystal with the magnetic field along *a* and *c* axes. The magnetic ordering of Tb and Mn atoms results in the anomaly of ΔL/L and giant MCE near $T_{{\text{C}}}^{{{\text{Tb}}}}$. A magnetic field-induced metamagnetic transition is observed along *c* axis above $T_{{\text{C}}}^{{{\text{Tb}}}}$, which leads to the magnetostriction effect and the plateau behavior of the MCE over a wide temperature range. The calculated results of $- \Delta S_{M}^{\max }$, RCP, and TEC(10) with the values of 24.02 J kg^−1^ K^−1^, 378.4 J kg^−1^, and 21.39 J kg^−1^ K^−1^ indicate that TbMn_2_Ge_2_ single crystal would be a promising candidate material for cryomagnetic refrigeration. Moreover, a *μ*_0_H-T phase diagram is established based on the magnetic behavior. The present results may provide some clue for searching novel magnetic refrigeration materials, and more studies need to be carried out to achieve a trade-off between the performance and cost, e.g., doping with light rare earth elements. Meanwhile, the temperature range where the MCE occurs needs to be further increased.

## Supplementary Information

Supplementary Figures.

## Data Availability

The datasets generated and/or analysed during the current study are available in the [Crystallography Open Database] repository, [3000405].
