# Supplementary material for: Anisotropic magnetic property, magnetostriction, and giant magnetocaloric effect with plateau behavior in TbMn2Ge2 single crystal
Source: Sci Rep. 2022 Nov 4;12:18727. doi: 10.1038/s41598-022-23661-4 (PMC9636430; doi:10.1038/s41598-022-23661-4)
Supplement: Supplementary file 1 — Supplementary Figures. [file 41598_2022_23661_MOESM1_ESM.docx]

**Supplementary Material**

**Anisotropic magnetic property,** **magnetostriction, and giant magnetocaloric effect with plateau behavior in TbMn_2_Ge_2_ single crystal**

Shuai Huang^1,3^*, Yuming Bai^2^, Kaiqi Wan^1^, Changming Zhu^1,3^, Dexuan Huo^1^, and Zhaoming Tian^2^*

^1^*Key Laboratory of Novel Materials for Sensor of Zhejiang Province, Institute of Material Physics, Hangzhou Dianzi University, Hangzhou 310018, P. R. China*

^2^*School of Physics and Wuhan National High Magnetic Field Cent, Huazhong University of Science and Technology, Wuhan 430074, P. R. China*

^3^*Guangxi Key Laboratory of Nuclear Physics and Nuclear Technology, Guangxi Normal University**, Guilin 541004, P. R. China*

*Corresponding author.

huangshuai@hdu.edu.cn (Shuai Huang).

tianzhaoming@hust.edu.cn (Zhaoming Tian).


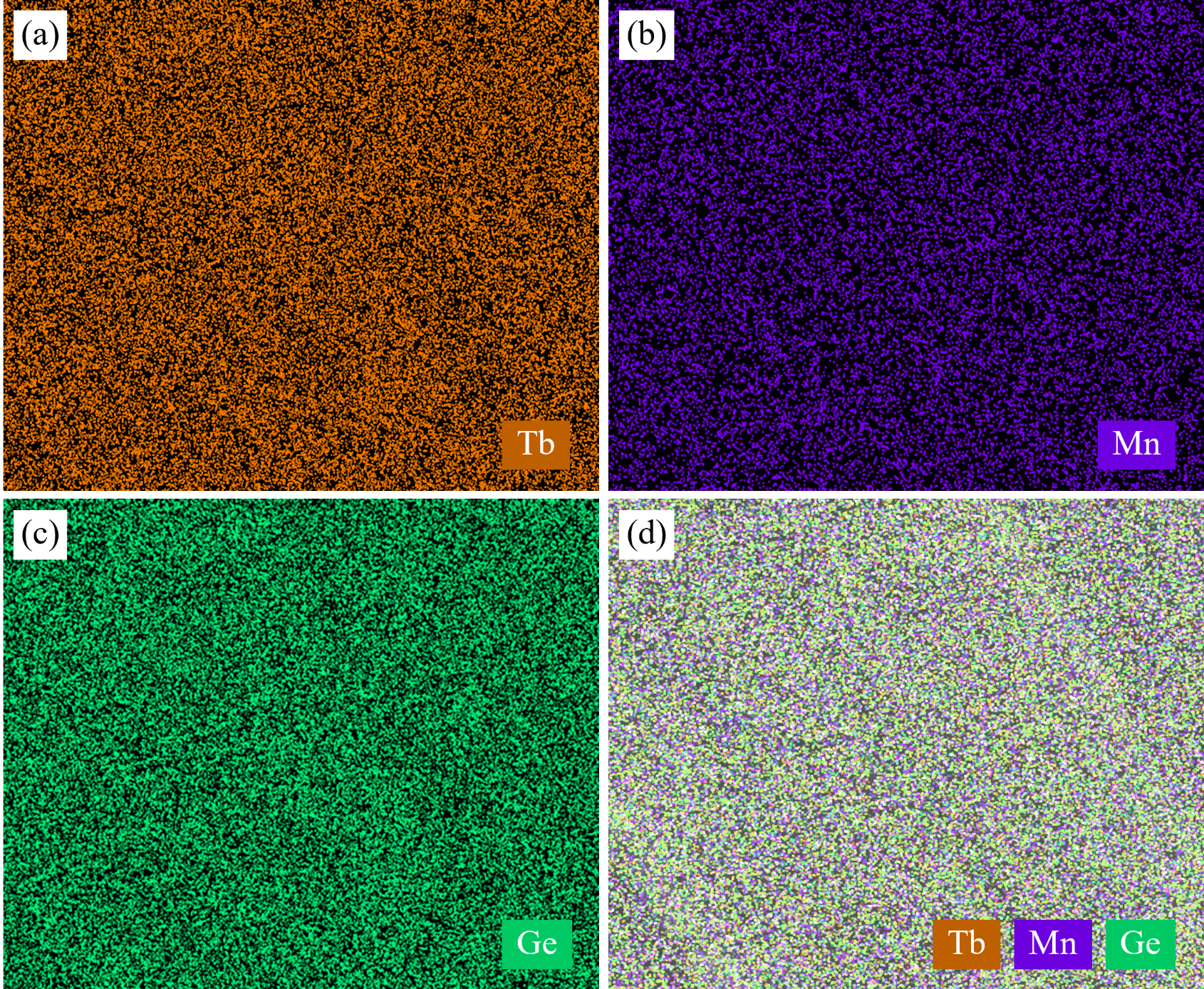


Fig. S1 The individual EDS image for (a) Tb, (b) Mn, (c) Ge, and (d) the combined Tb, Mn, and Ge EDS image for TbMn_2_Ge_2_ single crystal on a selected area.


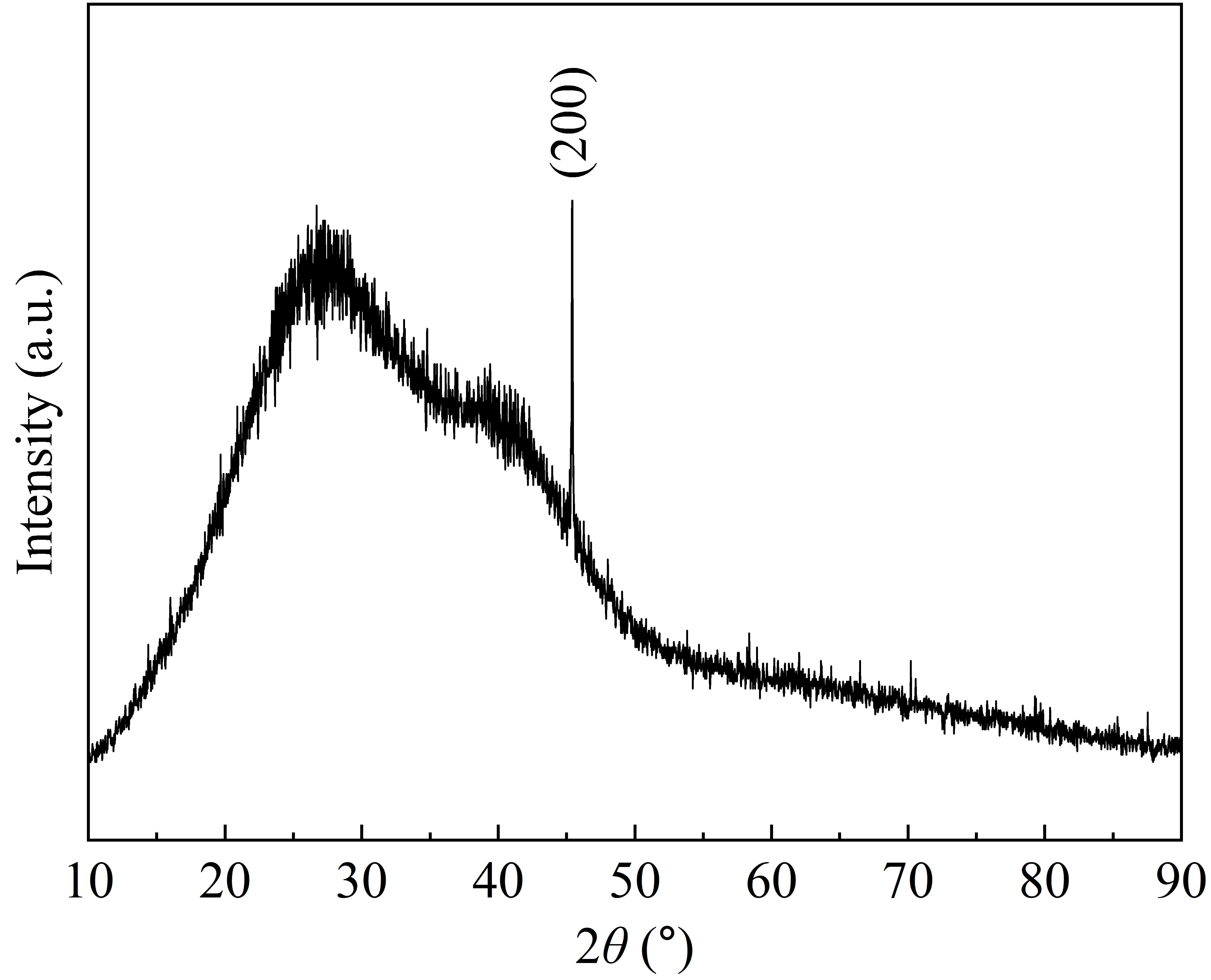


Fig. S2 Room-temperature XRD patterns for TbMn_2_Ge_2_ single crystal recorded on (*h*00) plane.


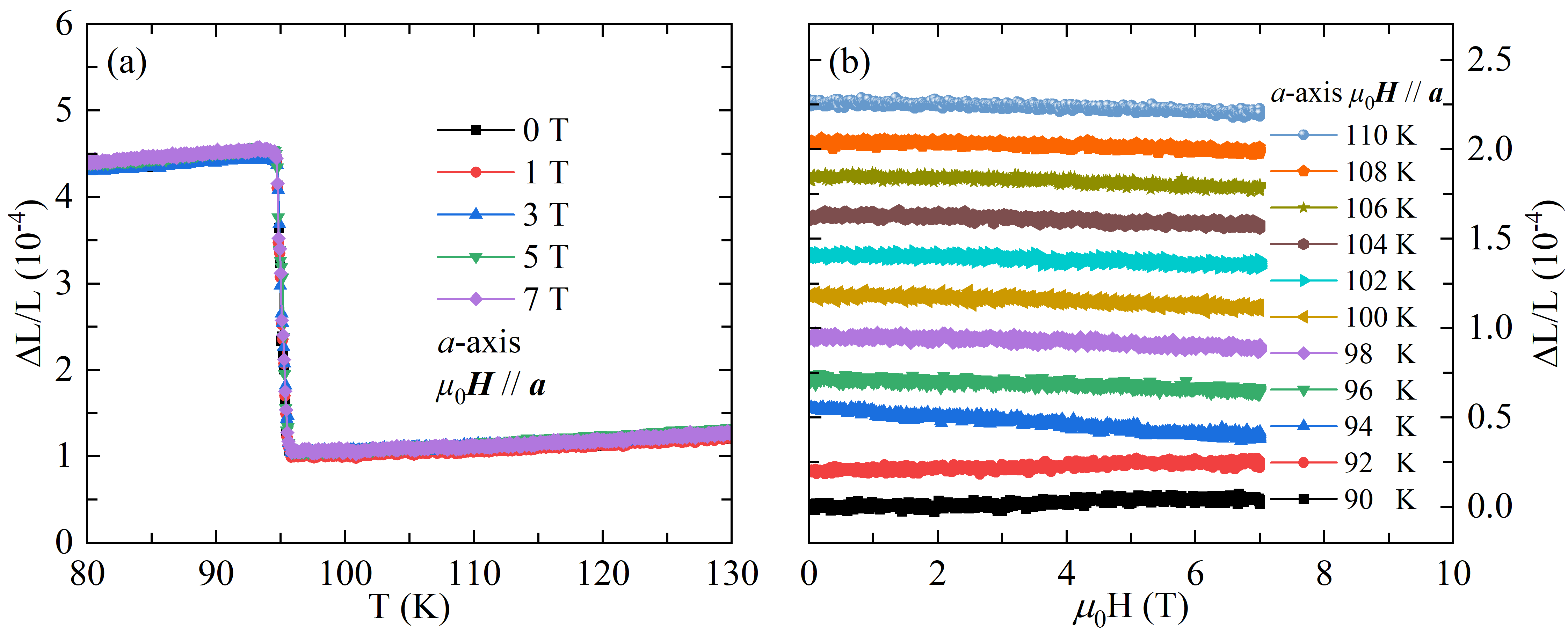


Fig. S3 (a) Temperature dependence of the strain change along *a* axis with *μ*_0_*H*//*a*. (b) Magnetic field dependence of magnetostriction [ΔL/L = ΔL/L(H) − ΔL/L(0)] along *a* axis with *μ*_0_*H*//*a* at different temperatures. For clarity, the curves are shifted upward except for the result with T = 90 K.
